# Adenosine A_2A_ Receptor: A Target for Regulating Renal Interstitial Fibrosis in Obstructive Nephropathy

**DOI:** 10.1371/journal.pone.0060173

**Published:** 2013-04-09

**Authors:** Hang Xiao, Hai-Ying Shen, Wei Liu, Ren-ping Xiong, Ping Li, Gang Meng, Nan Yang, Xing Chen, Liang-Yi Si, Yuan-Guo Zhou

**Affiliations:** 1 Department of Geriatrics, Southwest Hospital, Third Military Medical University, Chongqing, China; 2 Molecular Biology Center, State Key Laboratory of Trauma, Burn, and Combined Injury, Research Institute of Surgery and Daping Hospital, Third Military Medical University, Chongqing, China; 3 Department of Ophthalmology, Research Institute of Surgery and Daping Hospital, Third Military Medical University, Chongqing, China; 4 Department of Pathology, Southwest Hospital, Third Military Medical University, Chongqing, China; INSERM, France

## Abstract

Renal interstitial fibrosis (RIF) is the common pathological process of chronic kidney diseases leading inevitably to renal function deterioration. RIF and its preceding epithelial-mesenchymal transition (EMT) are commonly triggered by an early occurring renal inflammation. However, an effective approach to prevent EMT and RIF is still lacking and of urgent need. Recently, the adenosine A_2A_ receptor (A_2A_R) emerges as a novel inflammation regulator, therefore manipulation of A_2A_R may suppress the EMT process and as such protect against RIF. To test this hypothesis we applied a unilateral ureteral obstruction (UUO) model of RIF on A_2A_R knockout mice and their wild-type littermates, combined with the intervention of a selective A_2A_R agonist, CGS 21680. On days 3, 7 and 14 post-UUO we evaluated the effects of A_2A_R manipulation on the molecular pathological progresses of RIF, including the cellular component of interstitial infiltration, expression of profibrotic factors, cellular biomarkers of EMT, and collagen deposition of extracellular matrix. Our data demonstrated that activation of A_2A_R significantly suppressed the deposition of collagen types I and III, reduced the infiltration of CD4+ T lymphocytes, and attenuated the expression of TGF-β1 and ROCK1, which in turn inhibited and postponed the EMT progress. Conversely, genetic inactivation of A_2A_R exacerbated the aforementioned pathological processes of UUO-induced RIF. Together, activation of A_2A_R effectively alleviated EMT and RIF in mice, suggesting A_2A_R as a potential therapeutic target for the treatment of RIF.

## Introduction

Regardless of the etiology, almost all forms of end-stage renal disease share the common pathological feature of progressive renal interstitial fibrosis (RIF) and tubular atrophy [1], [2], [3]. Renal inflammation after sustained injuries, e.g. IgA nephropathy and lupus nephritis, serves as a primer that sets up the fibrogenic stage and triggers tissue fibrogenesis [4]. During this pathological progress macrophage and lymphocyte play crucial roles. RIF is characterized by the myofibroblast activation and the accumulation of matrix proteins including collagen types I (Col I) and type III (Col III). While RIF is commonly triggered by inflammatory processes recent studies suggest that a succedent epithelial-mesenchymal transition (EMT) may also play an important role in the progress of RIF [5]. Particularly, myofibroblast, with identified expression of α-SMA may contribute as a major source of increased production of matrix protein [6], [7]. Nevertheless, an early initiated anti-inflammatory strategy is therefore of importance to prevent the progression of RIF. However, no therapeutic approach is currently available to achieve this goal [8], [9]. Therefore, exploring new therapeutic target is in urgent need.

Recently, adenosine A_2A_ receptor (A_2A_R) emerges as a novel inflammation regulator affecting the inflammation process and tissue repair. Pharmacology studies showed that A_2A_R agonist, CGS21680 and ATL193, can effectively suppress inflammation [10], [11]. Activation of A_2A_R leads to attenuation of glomerulonephritis and renal injury [12], [13], [14]. Further, recent studies identified that A_2A_R activation inhibits Rho/ROCK1 in hepatic stellate cells [15]. All of the above strongly suggest that A_2A_R manipulation plays an important regulatory role in inflammation and may also affect EMT event. Therefore, we hypothesize that activation of A_2A_R may suppress cellular infiltration, EMT event and profibrogenic factors, thereby preventing consequent pathology of RIF. Conversely, inactivation of A_2A_R may lead exacerbation of RIF.

A unilateral ureteral obstruction (UUO) model has been refined to elucidate the pathogenesis and mechanisms responsible for RIF [16], [17]. It has been shown that the infiltration of macrophages and T cells and lymphocyte dysfunction are two major mechanisms contributing to the UUO-induced RIF model [18], [19]. In this model, at the cellular level, tubular dilatation leads the tubular epithelia to lose their epithelial characteristics and acquire mesenchymal traits such as α-SMA expression and actin reorganization. At molecular level, TGF-β1 plays a key role in EMT via activation of its downstream Rho/ROCK signaling pathway [20].

Using the experimental UUO-induced RIF mouse model, the present study was aimed to evaluate the modulatory effect of A_2A_R-based manipulation on several aspects of RIF progression, including interstitial lymphocyte infiltration, cellular biomarkers of EMT, expression of the profibrogenic factor TGF-β1 and its downstream Rho/ROCK1 pathway, as well as the consequent extracellular matrix accumulation.

## Materials and Methods

### Animals

All animal experiments were conducted under approval of the Institutional Animal Care and Use Committee of Third Military Medical University (TMMU) and performed under the supervision of the facility veterinary staff. Mice used in the present study, i.e., genetic A_2A_R knockout (KO) mice (A_2A_R^−/−^) and their wild-type (WT) controls (A_2A_R^+/+^) were bred at the Animal Care Center of the Research Institute of Surgery of TMMU after being imported from Boston University School of Medicine as previously described [21]. Mice were maintained in a pathogen-free, humidity- and temperature-controlled environment with 12 h light-dark cycles and free access to food and drinking water. The A_2A_R KO mice and their WT littermates were randomly designated into six experimental groups (see Table 1) according to the involvement of UUO procedure and CGS treatment. Ten mice from each experimental group were sacrificed at the designed experimental time-points, i.e., day 3, 7, and 14 after UUO. Mouse kidneys were harvested for the following imunohistochemistry evaluations.

**Table 1:** Experimental groups.

group	A_2A_R	UUO	CGS
WT+Sham	+	−	−
WT+UUO+Veh	+	+	−
WT+UUO+CGS	+	+	+
KO+Sham	−	−	−
KO+UUO+Veh	−	+	−
KO+UUO+CGS	−	+	+

### Unilateral ureteral obstruction (UUO) model

Mice (20–25 g weight) were subjected to the UUO procedure under anesthesia as previously described [22] with modifications. All surgical procedures were performed under an operating microscope. Briefly, mice were first anesthetized with sodium pentobarbital (50 mg/kg, i.p.). After a left flank incision was taken, the left ureter was exposed, ligated with 6–0 silk sutures at two points, and cut between the two ligatures. Lastly, the peritoneal membrane and skin were sutured. Sham surgery was performed as control by following all steps of UUO-procedure except ligation and cut of ureter.

### Drug treatment

Pharmacological activation of A_2A_R was induced by daily systemic administration of the selective A_2A_R agonist, CGS 21680 (Tocris, Cat# 1063, 0.4 mg/kg i.p.) from day 1 after UUO through the designed experimental time-points, i.e., day 3, 7, and 14 after UUO, when mice were scarified and their kidneys were harvested.

### Reverse transcription quantitative real-time PCR (RT-qPCR)

Total RNA extraction of renal sample was conducted using a total RNA extraction kit (BioFlux, Cat# BSC52S1) and the reverse transcription reaction was performed using SYBR Premix Ex Taq kit (DRR041A, Dalian, China), according to the manufacturer's instructions. Then qPCR was performed to quantify the expression level of A_2A_R, TGF-β1, and ROCK1 mRNAs using SYBR Premix Ex Taq kit (DRR041A, Dalian, China) and a qPCR reaction thermal cycle of 40 cycles of 95°C (30 s), 58°C (30 s), and 70°C (30 s). The glyceraldehyde 3-phosphate dehydrogenase (GAPDH) was used as an internal control to normalize RT-qPCR readout. Gene mRNA expression levels were calculated relative to the expression level of GAPDH. All primers used (as shown in Table 2) were synthetized by Takara (Dalian, China).

**Table 2:** Primers used for reverse transcription quantitative real-time PCR.

Gene	Primer	Sequence
A_2A_R	forward	5′-ccattcgccatcaccatcag-3′
	reverse	5′-cgtcaccaagccattgtacc-3′
ROCK1	forward	5′-acaccagaaggagctgaatgac-3′
	reverse	5′-ccgcaactgctcaatatcactc-3′
TGF-β1	forward	5′-tatttggagcctggacacacag-3′
	reverse	5′-cgtagtagacgatgggcagtg-3′

### Histopathology and Immunohistochemistry

Hematoxylin and eosin (H&E) staining and all immunohistostainings were performed on 4 μm-thick paraffin-embedded slice of kidney using a similar procedure as previously described [23]. The antigen retrieval process was performed by pressure cooking. The following primary antibodies and corresponding dilutions were used: anti-A_2A_R (ab115250, 1∶200, Abcam, Cambridge, MA USA), anti-CD3 (ab5690, 1∶100, Abcam), anti-CD4 (ab51312, 1∶100, Abcam), anti-CD8 (MA1-70041, 1∶50, Pierce), anti-Foxp3 (ab54501, 1∶200, Abcam), anti-CD11b (ab52478, 1∶100, Abcam), anti-CD68 (ab955, 1∶100, Abcam), anti-F4/80 (14-4801, 1∶50, Ebioscience), anti-collagen I (ab34710, 1∶400, Abcam), anti-collagen III (ab7778, 1∶400, Abcam), anti-α-SMA (ab7817, 1∶50, Abcam). All the quantitative morphological analyses were performed by separate investigator who was blinded to the treatment of samples. Positive stained cells and/or area were assessed and expressed as integrated optical density (IOD) or area. Three sections of each mouse kidney were measured, and 10 random fields or appointed area around vessels were chosen and calculated under magnification of 100x (for CD4) or 400x (for Col I, Col III and CD4+CD25+Foxp3+ Treg cell). The IOD or positive area was acquired by the Image-Pro Plus 6.0 program (Media Cybernetics, Bethesda, MD, USA).

### Western blot

The Western blot was performed according as previously described [24] with modification. Briefly, mouse kidneys were first homogenized in tissue protein extraction reagent (Thermo scientific, cat# MD156494) with a protease inhibitor cocktail (Thermo scientific, cat# ME156994) according to the manufacturer's instructions. Forty µg of protein extracts from each sample were loaded on and separated by 10% SDS-PAGE, then transferred onto nitrocellulose membrane. The blots were probed overnight at 4°C with primary antibodies against E-cadherin (ab76055, 1∶1000, Abcam), α-SMA (ab7817, 1∶200, Abcam), and β-actin (a2228, 1∶2000, Sigma-Aldrich), respectively, followed by the respective horseradish peroxidase-linked secondary antibody (a4416, 1∶5000, Sigma-Aldrich). Horseradish peroxidase activity was visualized via an enhanced chemiluminescence kit (20-500-120, Biolind, Israel). Images were scanned and processed for densitometric quantification by the Image analysis program (Labworks 4.0, UVP).

### Statistical analyses

The data are expressed as mean ± SD. Statistical analysis was performed using one-way ANOVA followed by Bonferroni *post hoc* comparisons. P<0.05 was considered statistical significance.

## Results

### 1. A_2A_R activation attenuated collagen deposition in matrix accumulation

**Figure 1:**
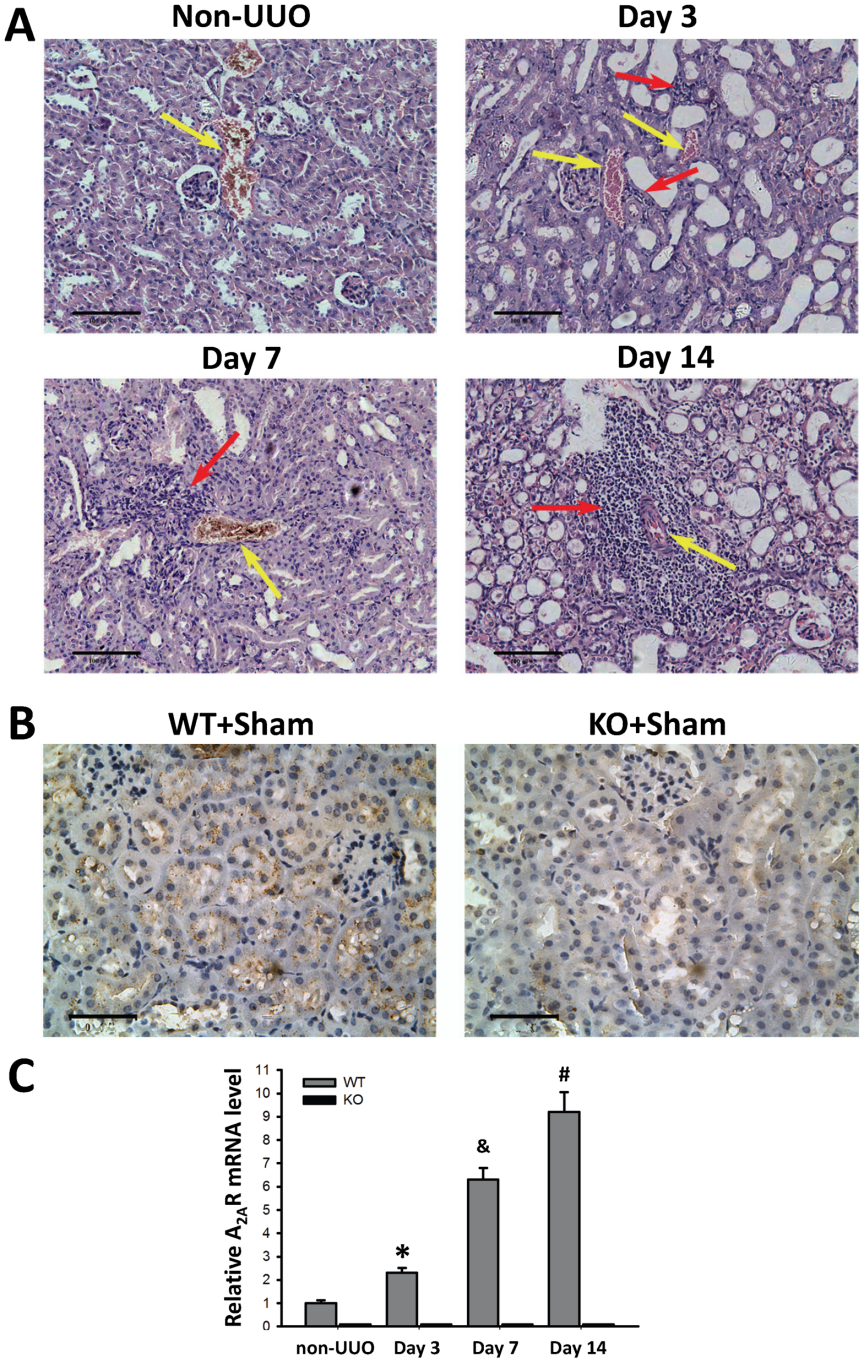
UUO-induced renal infiltration of leukocytes and increased mRNA expression of A_2A_R. (A) Representative H&E staining of renal tissue from mice subjected to UUO model. The infiltrations of leukocytes were observed around renal vessels (yellow arrow pointed), which were increased in a time-dependent manner, from day 0 through day 14 post-UUO. The red arrow is pointed at the inflammatory cells. Scale bar = 100 µm, 200×. (B) A_2A_R immunochemistry data showed positive stained renal tubular epithelial cells in wild-type mice (WT+Sham), but not in A_2A_R knockout mice (KO+Sham). Scale bar = 50 µm, 400x. (C) Demonstration of the renal levels of A_2A_R mRNA in non-UUO mice and at day 3, 7 and 14 post-UUO in mice. Data are mean ± SD. n = 10 per time point. * P<0.05, vs non-UUO WT mice; ^&^ P<0.05; vs WT day 3; ^#^ P<0.05, vs WT day 7, accordingly.

To evaluate the effect of A_2A_R on renal fibrosis, we applied the UUO model to mice combined with A_2A_R agonist CGS21680 and genetic A_2A_R inactivation (as aforementioned paradigm in Methods). Pathology assessment using H&E staining and immunohistostaining of Col I and Col III deposition were evaluated at day 3, 7, and 14 after UUO. Our H&E data demonstrated the successfulness of UUO modeling with featured pathological changes, e.g. progressively aggravated tubular dilatation and leukocytes infiltration (Figure 1 A). Our A_2A_R immunochemistry data demonstrated that positive stained renal tubular epithelial cells were seen in WT mice (WT+Sham), but devoid in KO mice (KO+Sham) (Figure 1B). Furthermore, we used RT-qPCR to detect the temporal changes of A_2A_R mRNA expression in the progress of UUO-induced RIF. We showed that the mRNA level of A_2A_R was significantly increased at day 3 through day 14 post-UUO, in a time-dependent manner. WT mice in WT+UUO+Veh group displayed an increase of 156%, 529% and 816% at day 3, 7 and 14, respectively, compared to non-UUO mice (F = 541.22, P<0.05, n = 10 per time point, Figure 1C). Conversely, A_2A_R mRNA level in A_2A_R KO (KO+UUO+Veh) mice remained under a detectable threshold from day 1 throughout day 14 post-UUO (Figure 1C).

**Figure 2:**
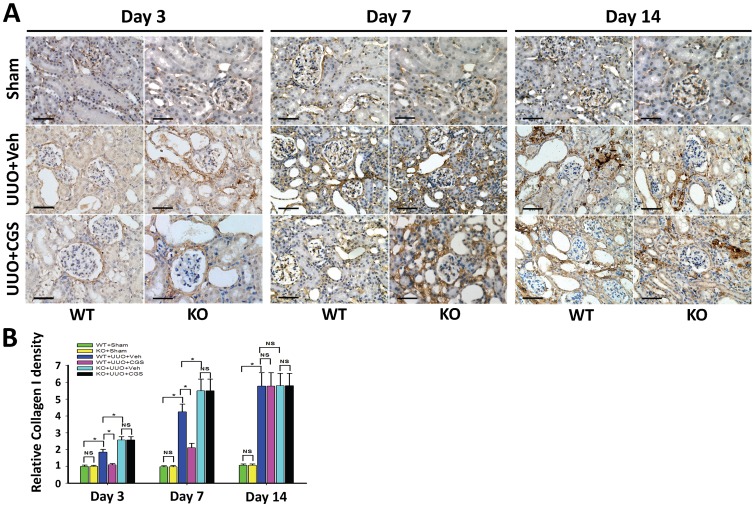
A_2A_R activity affected UUO-induced deposition of collagen I. (A) Representative immunohistochemistry of renal collagen I (Col I) from the A_2A_R KO and WT mice, at day 3, 7 and 14 post-UUO or sham surgery (Sham), following treatment of CGS21680 (CGS) or vehicle (Veh). Scale bar  = 50 µm, 400×. (B) Demonstration of Col I deposition in the post-UUO WT animals received treatment of vehicle (WT+UUO+Veh) or A_2A_R agonist CGS21680 (WT+UUO+CGS), and in the A_2A_R post-UUO KO mice received treatment of vehicle (KO+UUO+Veh), or CGS21680 (KO+UUO+CGS), at day 3, 7 and 14 post-UUO, along with that in sham control animals (WT+Sham and KO+Sham)(n = 10 per group). Data are mean ± SD. * P<0.05 between two compared groups; NS, no significance.

**Figure 3:**
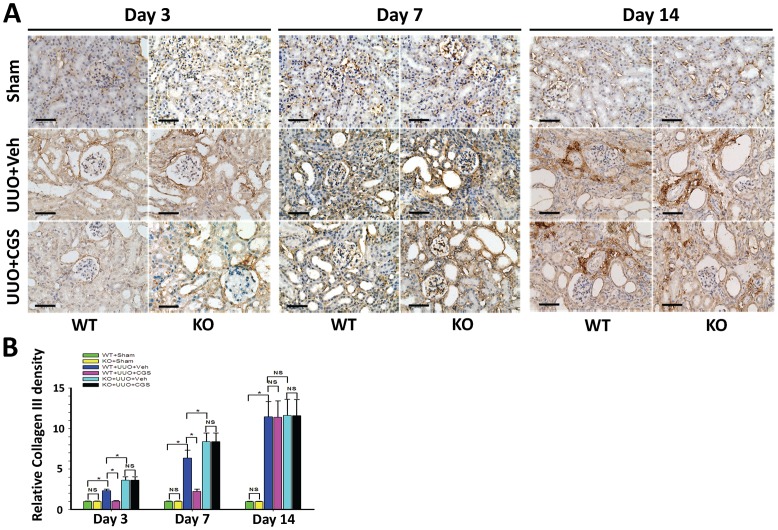
A_2A_R activity affected UUO-induced deposition of collagen III. (A) Representative immunohistochemistry of Collagen III) (Col III) from the A_2A_R KO and WT mice, at day 3, 7 and 14 post-UUO or Sham, following treatment of CGS21680 (CGS) or vehicle (Veh). Scale bar  = 50 µm, 400×. (B) Demonstration of Col III) deposition in the post-UUO WT animals received treatment of vehicle (WT+UUO+Veh) or A_2A_R agonist CGS21680 (WT+UUO+CGS), and in the A_2A_R post-UUO KO mice received treatment of vehicle (KO+UUO+Veh), or CGS21680 (KO+UUO+CGS), at day 3, 7 and 14 post-UUO, along with that in sham control animals (WT+Sham and KO+Sham)(n = 10 per group). Data are mean ± SD. * P<0.05 between two compared groups; NS, no significance.

Further, immunohistochemistry staining showed that starting at day 3, Col I and Col III progressively increased along with interstitial accumulation of extracellular matrix (ECM) in mouse kidneys from WT+UUO+Veh group (Figure 2 and 3), indicating the establishment of the UUO-induced RIF model. Importantly, quantitative morphometric analysis demonstrated that the depositions of Col I and Col III were both significantly reduced in the A_2A_R agonist-treated WT+UUO+CGS group (a reduction of 40.6% and 55.9% at day 3, a reduction of 50.3% and 64.9% at day 7, respectively), compared to WT+UUO+Veh group (P<0.05, n = 10 per groups, Figure 2 and 3). Conversely, genetic inactivation of A_2A_R significantly exacerbated collagen deposition in A_2A_R KO (KO+UUO+Veh) mice, showing an increased Col I and Col III levels (by 39.6% and 57.1% at day 3, 29.5% and 31.7% at day 7, vs WT+UUO+Veh group, respectively, P<0.05, n = 10 per groups, Figure 2 and 3). Noteworthy, genetic A_2A_R inactivation-induced exacerbation of collagen deposition was not affected by CGS treatment in KO+UUO+CGS group, showing significantly increased renal Col I and Col III levels, compared to WT+UUO+CGS group (P<0.05, n = 10 per groups, Figure 2 and 3).

Importantly, A_2A_R agonist CGS21680 treatment (in WT+UUO+CGS group) reversed deposition of collagens at day 3 and day 7 post-UUO, compared to WT+UUO+Veh group (P<0.05, vs n = 10 per groups, Figure 2 and 3). However, this inhibitory effect of CGS21680 was blunt at day 14 post-UUO, showing that the expression level of Col I and Col III in CGS21680-treated (WT+UUO+CGS) group were similar to that in other groups (P>0.05, n = 10 per group, Figure 2 and 3). Together, A_2A_R activation by CGS21680 resulted in suppression of collagen deposition at early post-UUO stage, i.e., at day 3 and day 7, but not at later post-UUO stage (day 14). Nevertheless, activation of A_2A_R effectively attenuated and postponed the progression of RIF whereas inactivation of A_2A_R exacerbated the RIF process.

### 2. A_2A_R activation inhibited UUO-induced changes on E-cadherin and SMA

**Figure 4:**
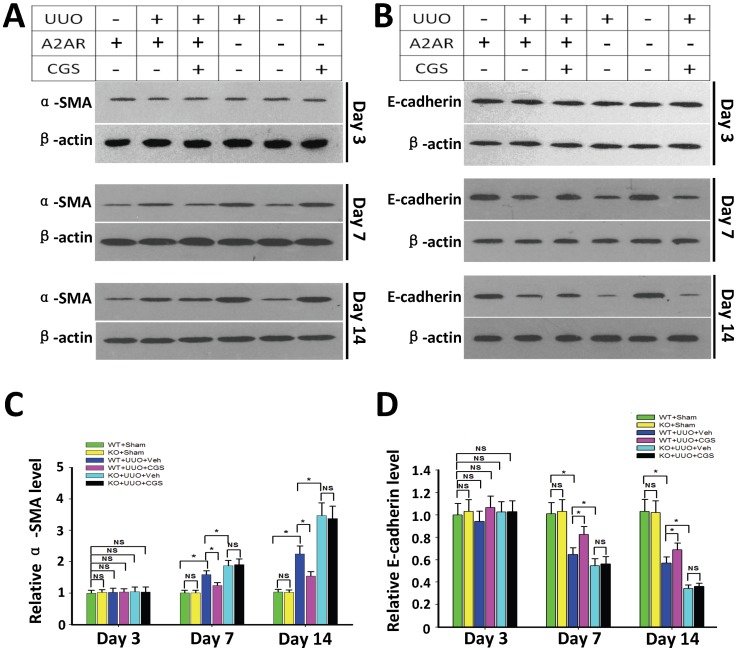
A_2A_R activity regulated UUO-induced expression of α-SMA and E-cadherin. (A, B) Representative Western blot of α-SMA (A) and E-cadherin (B) in post-UUO kidneys. (C, D) Demonstration of the expression level of α-SMA and E-cadherin in the sham (WT+sham and KO+sham) control mice and animals subjected to UUO with CGS21680 treatment (WT+UUO+CGS and KO+UUO+CGS) or with vehicle treatment (WT+UUO+Veh and KO+UUO+Veh), at day 3, 7 and 14 post-UUO (n = 5 per group). Data are expressed as mean ± SD. *P<0.05 between the two groups. NS, no significance.

To evaluate A_2A_R-mediated effects on the EMT process we detected the expression levels of α-SMA (the myofibroblast marker) and E-cadherin (the epithelial marker) that indicate the transdifferentiation status of epithelial to myofibroblast. Western blot assay showed that at day 3 no expression difference of α-SMA and E-cadherin was found between each of the UUO groups and sham groups (P>0.05, n = 5 per group, Figure 4), indicating the absence of EMT process. Notably, the expression level of α-SMA was enhanced by 58.6% at day 7, and 125.2% at day 14 in WT+UUO+Veh group compared to WT+Sham group (P <0.05, n =  5 group, Figure 4). However, the expression level of E-cadherin was reduced by 35.4% at day 7 and 43.0% at day 14 in WT+UUO+Veh groups compared to WT+Sham group (P<0.05, n = 5 per group, Figure 4). Importantly, A_2A_R agonist treatment reduced α-SMA level in WT+UUO+CGS group (by 21.7% at day 7 and 31.3% at day 14) compared to WT+UUO+Veh group, P<0.05, n = 5 per group, Figure 4). Meanwhile, A_2A_R agonist treatment enhanced E-cadherin level in WT+UUO+CGS group (by 27.9% at day 7 and by 20.6% at day 14) compared to WT+UUO+Veh group (P<0.05, day 7 and day 14, n = 5 per group, Figure 4). Conversely, inactivation of A_2A_R (KO+UUO+Veh) led to an opposite effect on α-SMA and E-cadherin levels compared to A_2A_R activation (WT+UUO+CGS) treatment. The expression of α-SMA was enhanced by 17.9% (day 7) and 54.2% (day 14), whereas the E-cadherin levels were decreased by 15.7% (day 7) and 39.6% (day 14), compared with WT+UUO+Veh group, (P<0.05, day 7 and day 14, n = 5 per group, Figure 4).

**Figure 5:**
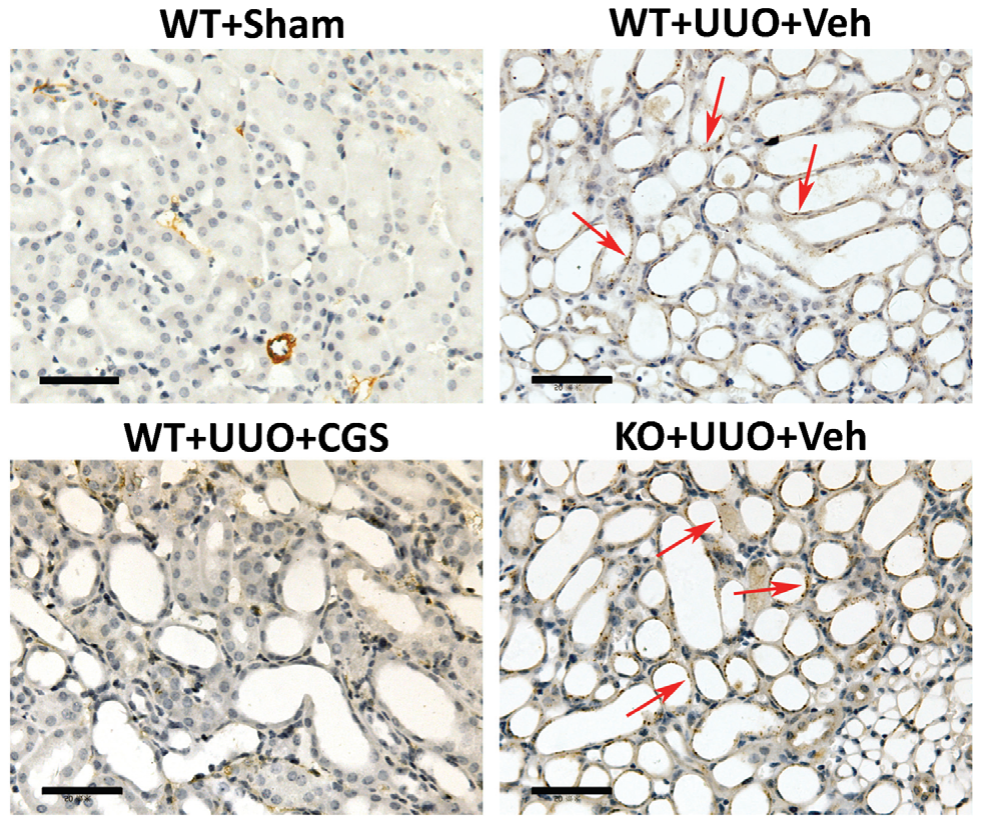
A_2A_R activation inhibited UUO-induced EMT process. Representative immunohistochemistry staining of α-SMA in mice at day 7 post-UUO. The α-SMA, as the marker for myofibroblast (red arrow), was positively stained on the renal tubular epithelial cells in WT (WT+UUO+Veh) and A_2A_R KO (KO+UUO+Veh) mice whereas treatment of CGS21680 reduced positive staining of α-SMA in WT+UUO+CGS mice. Scale bar  = 50 µm, 400x.

In addition, our immunochemistry data demonstrated that positive stained renal tubular epithelial cells were seen in vehicle-treated WT mice (WT+UUO+Veh) and A_2A_R KO mutants (KO+UUO+Veh), but devoid in WT mice which received CGS21680 treatment (WT+UUO+CGS), at day 7 post-UUO (Figure 5). This immunohistology data is consistent with our Western blot evaluations of α-SMA. Together, A_2A_R activation-induced reduction of α-SMA and the increase of E-cadherin suggest an inhibitory effect of A_2A_R on the tubular EMT process.

### 3. A_2A_R activation attenuated the expression of profibrotic mediators

**Figure 6:**
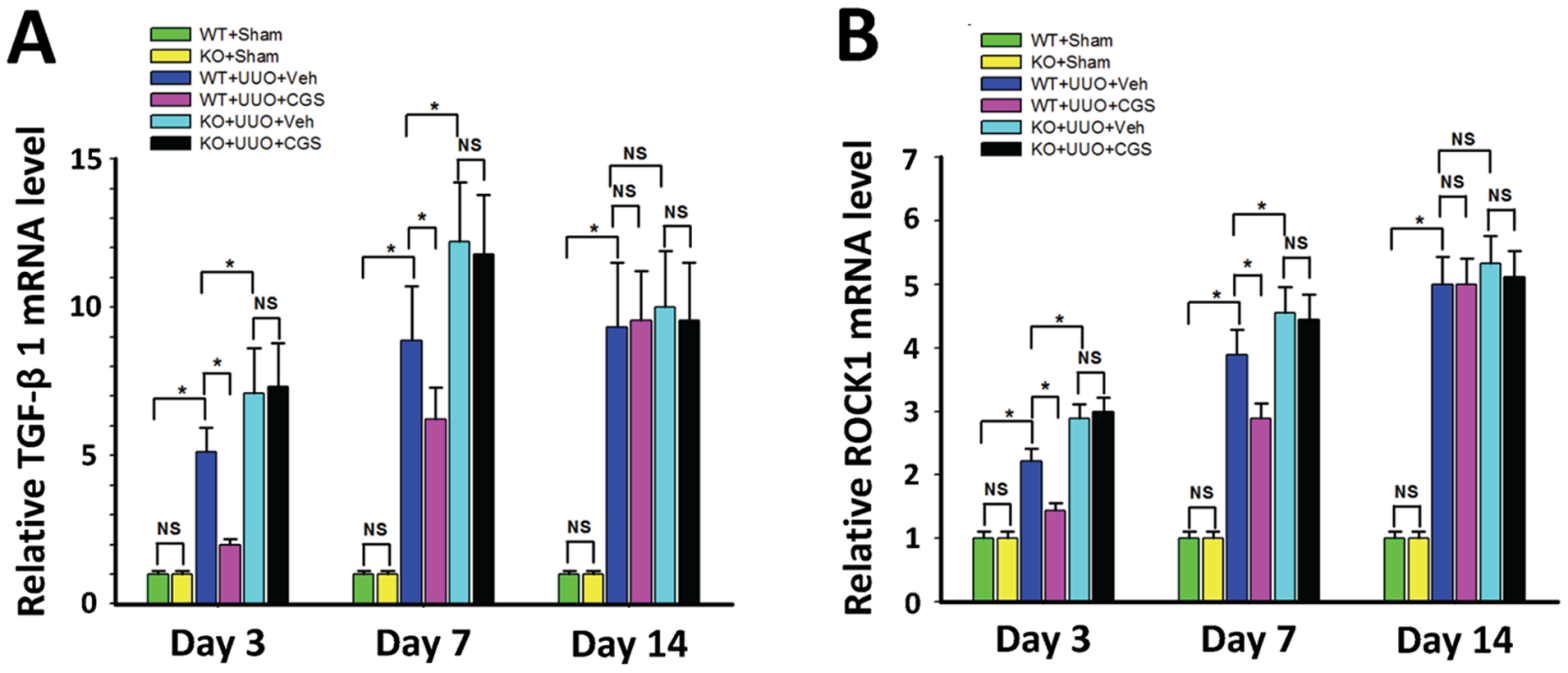
A_2A_R activity affected UUO-induced mRNA expression of TGF-β1 and ROCK1. Demonstration of the mRNA expression levels of TGF-β1 (A) and ROCK1 (B) in the sham (WT+sham and KO+sham) control mice and animals subjected to UUO with CGS21680 treatment (WT+UUO+CGS and KO+UUO+CGS) or with vehicle treatment (WT+UUO+Veh and KO+UUO+Veh), at day 3, 7 and 14 post-UUO. (n = 10 per group). Data are mean ± SD. *P<0.05, between compared groups; NS, no significance.

To mechanistically evaluate the A_2A_R modulation on RIF, we detected the mRNA expression of two crucial profibrotic mediators, TGF-β1 and ROCK1 using RT-qPCR. We showed that the expression level of TGF-β1 mRNA was significantly increased at day 3 through day 14 in WT+UUO+Veh group (an increase of 411%, 789% and 833% at day 3, 7 and 14 respectively) compared to WT+Sham control group (P<0.05, n = 10 per group, Figure 6). Importantly, A_2A_R agonist treatment attenuated the increase of TGF-β1 mRNA expression in WT+UUO+CGS group, leading to a decrease of 60.9% (P<0.05) and 30.0% (P<0.05) at day 3 and day 7, respectively, vs. WT+UUO+Veh group (n =  10 per group). Conversely, genetic inactivation of A_2A_R in KO+UUO+Veh group led to an additional enhancement in mRNA expression of TGF-β1, by 39.1% (day 3) and 37.5% (day 7) compared to WT+UUO+Veh group, correspondingly (P<0.05, n = 10 per group, Figure 6). Noteworthy, A_2A_R activation-mediated inhibitory effect on TGF-β1 expression was blunt at 14 day post-UUO, with no difference compared to other UUO groups (P>0.05 Figure 6), suggesting that the A_2A_R activation-induced suppression on TGF-β1 expression occurred at early but not later post-UUO stage.

Furthermore, RT-qPCR data showed that the expression level of ROCK1 mRNA was significantly enhanced in kidneys from WT+UUO+Veh mice, leading to an increase of 122%, 289% and 400%, at day 3, 7, and 14, correspondently, compared to WT+Sham group (P<0.05, n = 10 per group, Figure 6). The increase of ROCK1 mRNA shared a similar post-UUO expression pattern of TGF-β1 mRNA. Importantly, the increased expression of ROCK1 was suppressed in CGS21680-treated WT+UUO+CGS animals, showing a reduction of 35.0% (day 3) and 25.7% (day 7) vs. WT+UUO+Veh (P<0.05, n = 10 per group, Figure 6). In contrast, genetic inactivation of A_2A_R (in KO+UUO+Veh group) led to an exacerbated enhancement of ROCK1 level, by 30.0% (day 3) and 17.1% (day 7) vs. WT+UUO+Veh (P<0.05, day 3; P<0.05, day 7; n = 10 per group, Figure 6). Interestingly, the A_2A_R effect on ROCK1 expression was also noticed only at day 3 and day 7, but not on day 14, post-UUO (P>0.05, Figure 6). Together, these findings revealed that A_2A_R activation inhibited expression of TGF-β1 and its downstream factor, ROCK1.

### 4. Suppression on T lymphocyte infiltration contributes to A_2A_R-mediated renal protection against RIF

**Figure 7:**
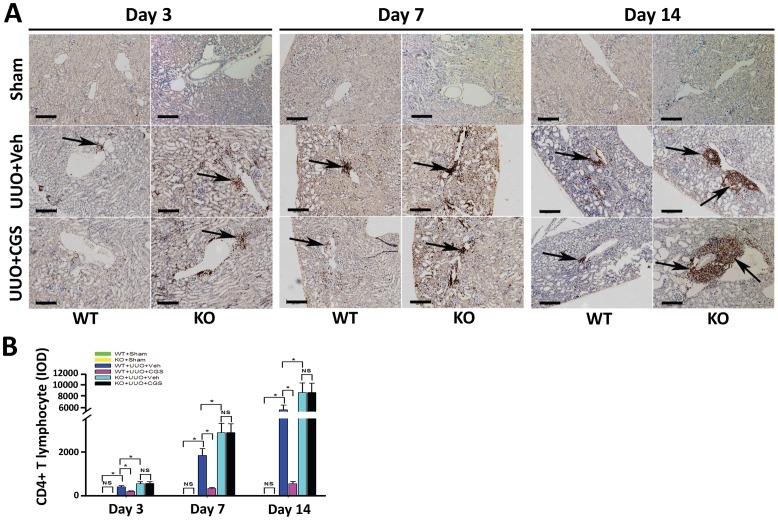
Immunohistochemistry stained for CD4+ T lymphocyte of kidney sections. (A) Representative immunohistochemistry staining of CD4 from the kidneys of A_2A_R KO and WT mice, at day 3, 7 and 14 post-UUO or Sham following treatment of CGS21680 (CGS) or vehicle (Veh). CD4+ T lymphocyte infiltration was prominent around vessels (black arrow pointed). (B) Demonstration of CD4+ T lymphocyte staining at day 3, 7 and 14 post-UUO in the sham, (WT+sham and KO+sham) control mice and animals subjected to UUO with CGS21680 treatment (WT+UUO+CGS and KO+UUO+CGS) or with vehicle treatment (WT+UUO+Veh and KO+UUO+Veh). (n = 10 per group). Scale bar = 200 µm, 100x. Data are mean ± SD. * P<0.05 between compared groups.

Infiltration of T lymphocyte, a key cellular inflammatory response, plays a crucial role in the initiation of EMT and RIF. Thus we examined renal T lymphocyte infiltration post-UUO using immunostaining of T lymphocyte cell marker, CD3, CD4 and CD8. We observed that a prominent accumulation of CD3-positive stained (CD3+) T lymphocyte was located around vessels in the kidneys from WT+UUO+Veh animals (data not shown). To identify the subtype of CD3+ T lymphocytes, we preformed immunohistostaining of CD4 and CD8. The data showed that the infiltrating T lymphocytes in UUO groups were identified as CD4-positive stained (CD4+) (Figure 7), but not CD8 positive (CD8+) cells (data not shown). Further, quantitative morphometric analysis demonstrated that in WT+UUO+CGS mice there was less infiltration of CD4+ T lymphocyte, showing a reduction of 51.5% (day 3), 82.4% (day 7) and 89.9% (day 14) correspondently, compared with WT+UUO+Veh animals (P <0.05, n = 10 per group, Figure 7). Conversely, infiltration of CD4+ T lymphocyte was exacerbated in UUO mice with genetic inactivation of A_2A_R (KO+UUO+Veh), showing an increase of 37.8% (day 3), 57.5% (day 7), and 61.2% (day 14), respectively, vs WT+UUO+Veh group (P<0.05, n = 10 per group, Figure 7). In addition, immunohistostaining of CD11b (a marker for neutrophil granulocyte) as well as CD68 and F4/80 (markers for macrophage) were also performed to detect the involvement of other inflammatory cellular components. However, positive staining of CD68, F4/80, and CD11b were observed without significant difference (data not shown), suggesting a devoid of infiltration of macrophage or neutrophil granulocyte.

**Figure 8:**
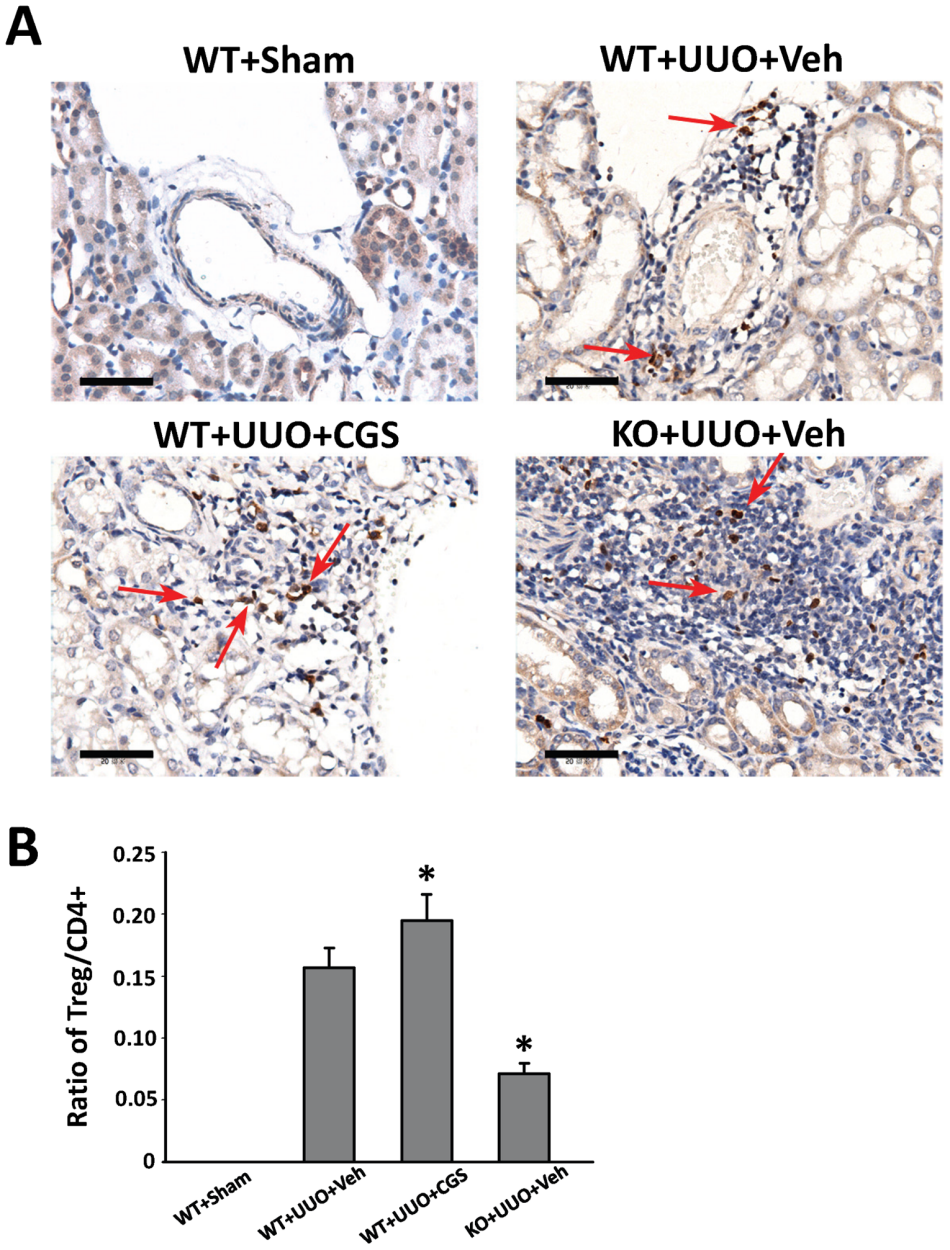
Immunohistochemistry stained for CD4+CD25+ Foxp3+ Treg of kidney sections. (A) Representative immunohistochemistry staining of Foxp3 of mice subjected to the UUO modeling. (B) Demonstration the ratio of Treg to CD4+ T lymphocytes at day14 in the sham (WT+sham) control mice and animals subjected to UUO with CGS21680 treatment (WT+UUO+CGS) or with vehicle treatment (WT+UUO+Veh and KO+UUO+Veh). n = 10 per group. *P<0.05 vs UUO+Veh group. Scale bar = 50 µm; 400×.

Lastly, we performed immunohistochemistry staining of Foxp3 (a marker of T cell), to evaluate the involvement of CD4+CD25+Foxp3+ regulatory (Treg) cells that are important inflammation regulators. We demonstrated the presence of Treg cells in all UUO groups at day 14 (Figure 8). Further, the quantitative morphometric analysis showed that the ratio of Foxp3+ Treg cells to CD4+ T lymphocytes was enhanced 24.2% in WT+UUO+CGS animals (n = 10 per group), whereas genetic A_2A_R inactivation significantly decreased this ratio by 54.8% in KO+UUO+Veh group, compared with WT+UUO+Veh group (P<0.05, n = 10 per group) at day 14 post-UUO. Together, these findings suggest that CD4+ T lymphocyte was the major component in the inflammatory infiltration after UUO while A_2A_R-activation suppressed CD4+ T lymphocyte infiltration and enhanced the proportion of Treg cells.

## Discussion

Our study demonstrate, for the first time, that A_2A_R activation can protect and postpone RIF in experimental UUO animals by the following findings: (i) A_2A_R activation significantly attenuated UUO-induced pathology consequence and collagen deposition at early stage post-UUO; (ii) A_2A_R activation inhibited changes of E-cadherin and SMA – two EMT-related changes in RIF; (iii) A_2A_R activation attenuated the expression of profibrotic mediators TGF-β1 and its downstream Roh/ROCK1 pathway; (iv) Importantly, those effects were associated with A_2A_R-mediated suppression on infiltration of T lymphocyte. Conversely, inactivation of A_2A_R conducted an opposite effect in the above phenotypes. These findings demonstrated that activation of A_2A_R is of importance in phenotypic conversion of RIF, suggesting A_2A_R may become a potential therapeutic target against RIF.

Regulation of the infiltration of T lymphocytes is the principal mechanism of A_2A_R manipulation in UUO-induced RIF in mice. The infiltration of lymphocyte, as a macrophage-independent response, plays an important role in the process of RIF and nephritis [19], [25], [26], [27], [28]. T cell infiltration was observed in the kidneys of patient with chronic kidney disease [29] and in the models of UUO [30], [31], [32]. Furthermore, there is reduced lymphocyte infiltration and fibrosis in the kidney after UUO when CC-chemokine receptor-1 mediated migration of lymphocytes into inflamed tissue is blocked [31], [33]. Activation of A_2A_R, as a Gs coupling protein receptor, can significantly increase cAMP level in immune cells, and in turn, alter immune responses including antigen presentation, T cell activation, clonal expansion, and the survival of immune memory [34], [35]. This study shows that activation of A_2A_R significantly reduced the CD4+ T cell infiltration whereas genetic inactivation of A_2A_R exerted the opposite effect. Noteworthy, while macrophages played an important role in renal fibrosis in a model of immune-associated chronic inflammation [13] and in aristolochic acid-induced RIF [36], in the presented UUO model no significant amount of macrophage (with CD68+ and F4/80+ staining) was observed participating in leukocyte infiltration around vessels post-UUO.

Treg cells are critical to maintain immune-cell homeostasis by enforcing a dominant negative regulation on other immune cells. Thus, Tregs are of great interest due to its immunosuppressive effect and inhibitory effect against fibrosis [37]. Most recent, it was reported that Tregs' negative regulation on immune cells was mediated by A_2A_R activation whereas deletion of A_2A_R abolished Tregs' regulatory effect [38]. These reports support our findings that A_2A_R activation by CGS21680 significantly increased the ratio of Tregs to CD4+ T lymphocytes, whereas this ratio was significantly decreased in A_2A_R KO mutants post-UUO. Thus, regulation of Tregs recruitment and (CD4+) T lymphocyte infiltration acts as underlying mechanism of A_2A_R-mediated effects against RIF.

Another important finding in this study is that A_2A_R could affect EMT-related changes in E-cadherin and SMA. While more direct evidence and evaluations are needed in human studies, EMT is recently proposed as a crucial mechanism in RIF [5]. During the EMT process, renal tubular epithelial cell lost the E-cadherin phenotype and acquire the myofibroblast phenotype α-SMA. Our findings demonstrated that activation of A_2A_R restored expression level of α-SMA and E-cadherin to a basal level in sham animals (Figure 4). Though indirectly based on Western blot reflecting total renal tissue rather than TECs-specific on-site changes, this finding indicates that A_2A_R activation maintained intrinsic phenotypes of epithelia and myofibroblast, i.e., inhibited the process of EMT. To find the mechanism by which A_2A_R affects EMT, we demonstrated that activation of A_2A_R significantly reduced the expression of TGF-β1, a key profibrotic mediator in EMT, along with ROCK1, the regulatory protein in the TGF-β1 downstream pathway Rho/ROCK signaling. In UUO, the enhanced TGF-β1 may (i) act as a mitogenic factor to affect collagen synthesis and (ii) facilitate the EMT process [6], [39]. Importantly, activation of A_2A_R restored both aforementioned consequences of TGF-β1 post-UUO. Furthermore, this study showed that TGF-β1-mediated EMT is regulated by the Rho/ROCK-dependent signaling pathway [20], and the ROCK pathways play an important role in RIF and phenotypic modulation of epithelial cells [40], [41]. While ROCK has two types (ROCK1 and ROCK2), the ROCK1 is predominantly expressed in the kidney and regulates cell adhesion, chemotaxis and contraction, as well as epithelial differentiation [42]. Meanwhile, E-cadherin, not only as a marker, is also the most important component for maintaining the integrity and polarity of epithelial cells [43]. The loss of E-cadherin expression in the renal tubular epithelial cells will lead to a loss of cell-cell adhesion facilitating the renal tubular epithelial cells enter the renal interstitium. Studies showed that E-cadherin is regulated by ROCK1 [42], [44], moreover, α-SMA as the important structure protein of the myofibroblast, is also influenced by the Rho/ROCK signaling pathway [45]. Importantly, the Rho/ROCK-1 pathway is closely linked to adenosine activity [15]. Thus activation of A_2A_R may, via ROCK1, regulate cell adhesion of tubular epithelial cells and the EMT process. Further evidence is needed to address this potential mechanism.

The increase of A_2A_R after UUO may account for a compensatory protective mechanism. In line with our finding, Lee et al also demonstrated this phenomena [46]. However, it is still unclear whether the increased A_2A_R mRNA attribute to inherent renal cells or immigrated cells, e.g., bone marrow-derived cells [47], via inflammatory processes post-UUO. Our study suggested that the effect on T lymphocyte infiltration contribute to A_2A_R-mediated protection against RIF. Noteworthy, some of the A_2A_R activation resulting effects on post-UUO animals were blunt in the late stage after UUO with unknown reason. This may be due to the severity of phenotypes in late post-UUO stage and the progressive aggravation in pathology unless the pathogenic factors of tubular obstruction might have been removed. The severe pathology changes in late post-UUO stage might be irreversible; however, we also noted the suppressive effect of A_2A_R activation on expression of TGF-β1 was devoid at day 14 after UUO. This may be due to a redundant mechanism between ROCK1 and TGF-β1, for instance a down-regulation of ROCK1 by A_2A_R agonist may result in an increased expression of TGF-β1 [48]. To clarify the noticed phenomenon, using a model with mild severity or slow progress may help on this point in the future.

In summary, this study for the first time demonstrates the beneficial effect of A_2A_R activation in preventing the progression of RIF in the UUO animal model. This provides a novel therapeutic strategy against renal interstitial fibrosis by targeting the adenosine A_2A_ receptor.
