# Expression of Concern: Adenosine A_2A_ Receptor: A Target for Regulating Renal Interstitial Fibrosis in Obstructive Nephropathy

**DOI:** 10.1371/journal.pone.0314149

**Published:** 2024-11-14

**Authors:** The PLOS ONE Editors

After this article [1] was published, concerns were raised about Figs 2–4 and 6–7. Specifically,

- In Fig 2A, the Day 3 Sham KO panel and the Day 7 Sham KO panel appear similar.
- In the quantitative graph data in Figs 2, 3, 4, 6 and 7, the error bars for multiple conditions appear similar and proportional to the overall size of the bar.

In response to queries about the experiments in Fig 2A, the first author stated that the Day 3 Sham KO and Day 7 Sham KO panels were erroneously duplicated and the Day 3 KO Sham panel is incorrect. An updated version of Fig 2 is provided here and the corresponding underlying images taken at the time of the original experiments are in S1–S3 Files. New images taken from the original Day 3 Sham KO paraffin embedded blocks are provided in S4 File.

In response to queries about the graph data in Figs 2–4 and 6–7, the first author stated that all numerical values in Figs 2, 3, 4 and 6, including the error bars which represent the range of standard deviation around the mean, are presented as relative values, with the WT+SHAM group serving as the baseline (set at 1) for comparison with other groups. They also stated that for Fig 7, due to the large differences between groups, the mean and standard deviation of IOD were used. A statistical reviewer reviewed this response and noted that standard deviations do not provide enough information to draw between-group comparisons for the six groups. The statistical analysis and error bar concerns call into question the reliability of the quantitative results reported in the article.

The first author provided partial datasets for the graphs in Figs 4 and 6 and stated that the remainder of the data for this article [1] are no longer available with the exception of some of the underlying blots for Fig 4B (S5 File). Due to the file type, PLOS was unable to open the quantitative files provided.

The concerns with the quantitative results in Figs 2, 3, 4, 6 and 7 cannot be resolved due to the unavailability of the full quantitative datasets. Therefore, the *PLOS ONE* Editors issue this Expression of Concern.

**Fig 2:**
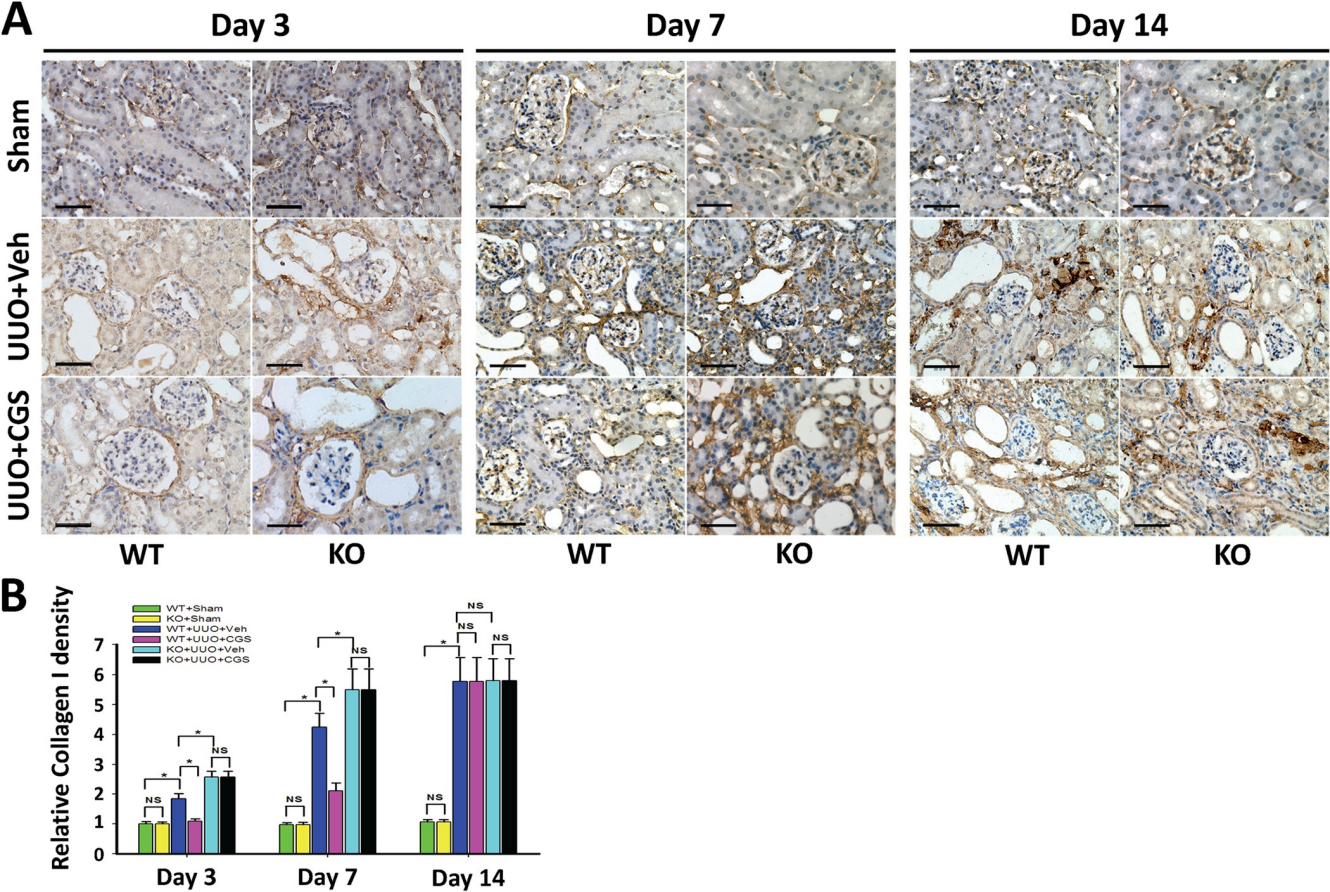
A_2A_R activity affected UUO-induced deposition of collagen I. (A) Representative immunohistochemistry of renal collagen I (Col I) from the A_2A_R KO and WT mice, at day 3, 7 and 14 post-UUO or sham surgery (Sham), following treatment of CGS21680 (CGS) or vehicle (Veh). Scale bar   =  50 µm, 400×. (B) Demonstration of Col I deposition in the post-UUO WT animals received treatment of vehicle (WT+UUO+Veh) or A_2A_R agonist CGS21680 (WT+UUO+CGS), and in the A_2A_R post-UUO KO mice received treatment of vehicle (KO+UUO+Veh), or CGS21680 (KO+UUO+CGS), at day 3, 7 and 14 post-UUO, along with that in sham control animals (WT+Sham and KO+Sham)(n  =  10 per group). Data are mean ± SD. * P<0.05 between two compared groups; NS, no significance.

## Supporting information

S1 File Original underlying images for Fig 2 (Day 3). (PDF)

S2 File Original underlying images for Fig 2 (Day 7). (PDF)

S3 File Original underlying images for Fig 2 (Day 14). (PDF)

S4 File New Day 3 Sham KO images from the paraffin original blocks. (PPTX)

S5 File Underlying blots supporting Day 3 E-cadherin and β-actin blots in Fig 4B. (JPG)
