# Supplementary figures and images for: Expression of Concern: Adenosine A2A Receptor: A Target for Regulating Renal Interstitial Fibrosis in Obstructive Nephropathy
Source: PLoS One. 2024 Nov 14;19(11):e0314149. doi: 10.1371/journal.pone.0314149 (PMC11563404; doi:10.1371/journal.pone.0314149)

Sham

UUO+Veh

UUO+CGS

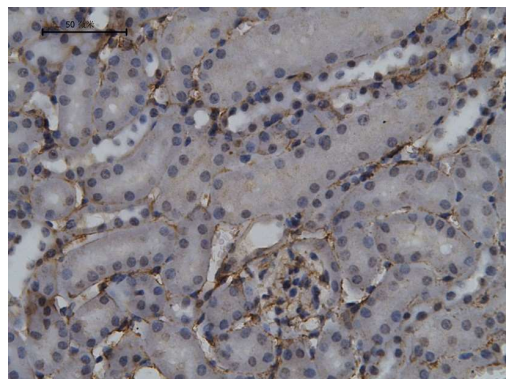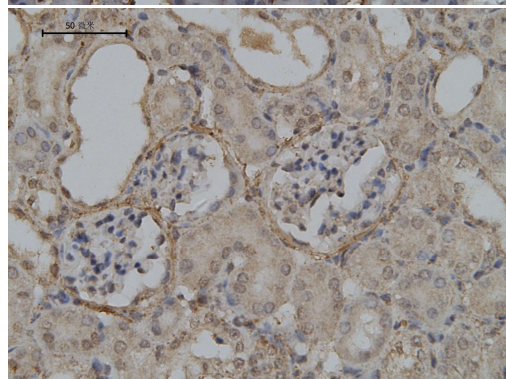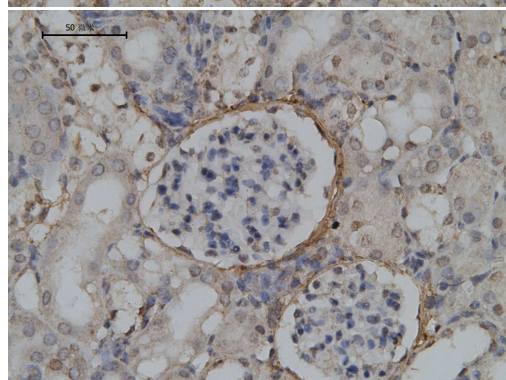

WT

Day 3

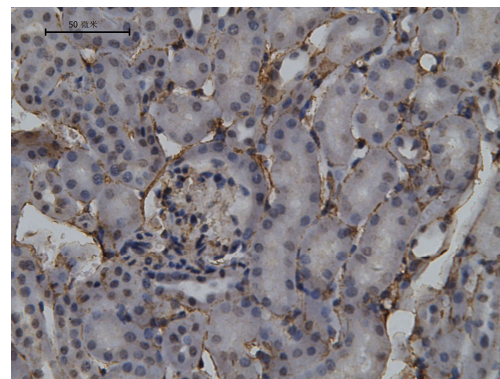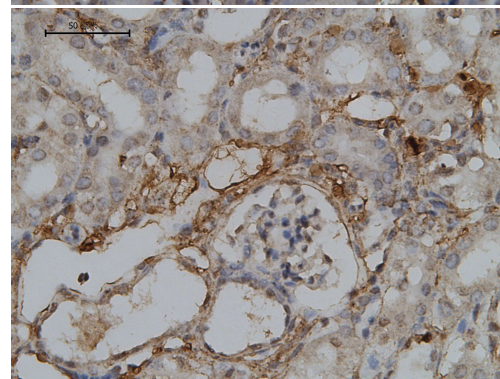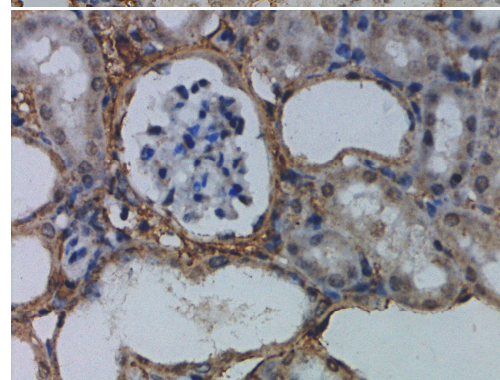

KO

Supplement: S1 File — (PDF) [file pone.0314149.s001.pdf]

WT      UUO+CGS      UUO+Veh      Sham

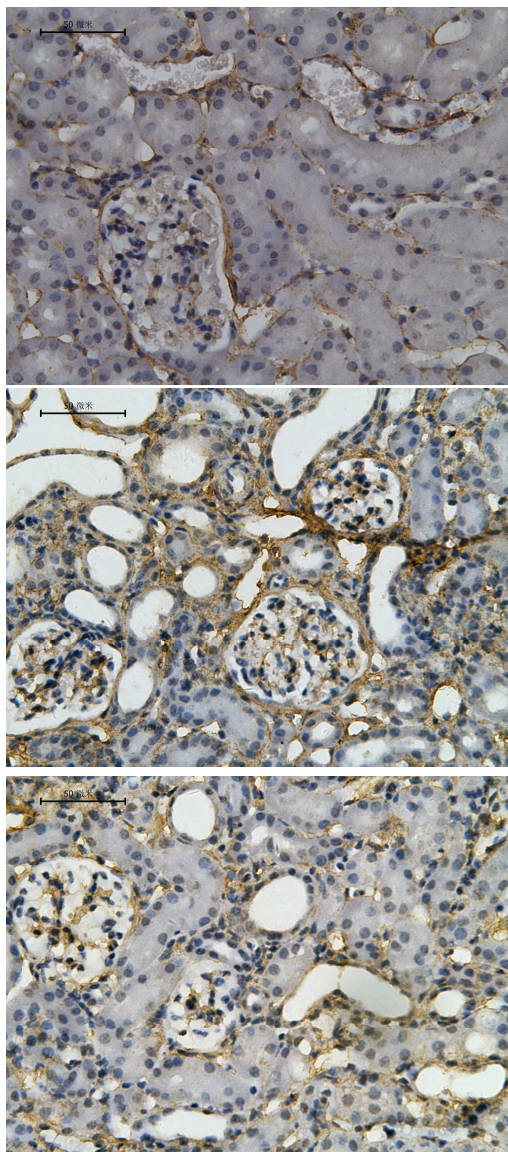

WT

Day 7

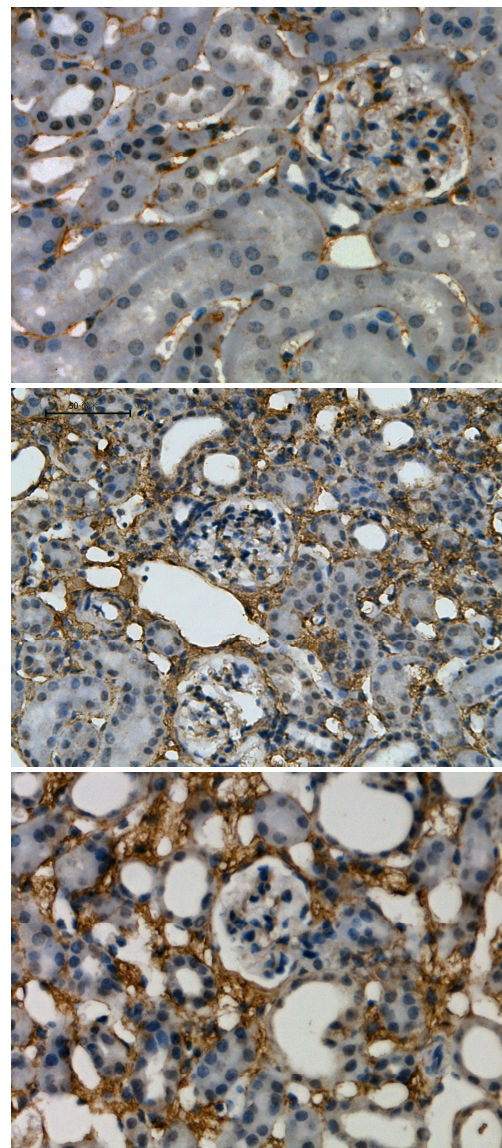

KO

Supplement: S2 File — (PDF) [file pone.0314149.s002.pdf]

Sham  
UO+Veh  
UO+CGS

Day 14

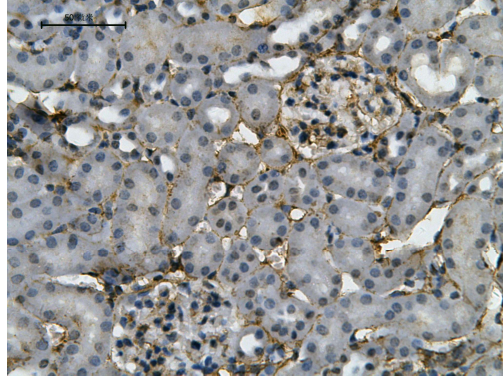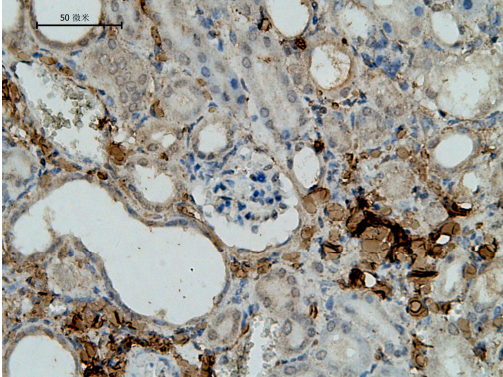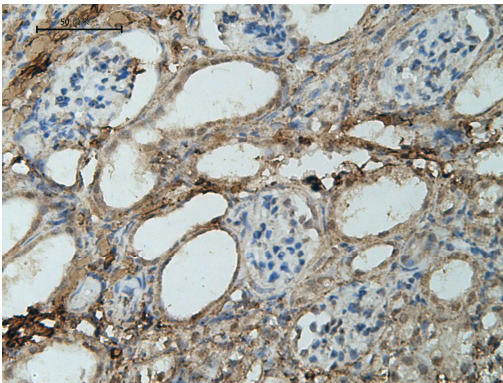

WT

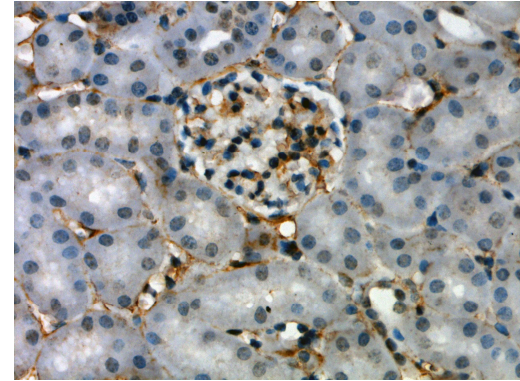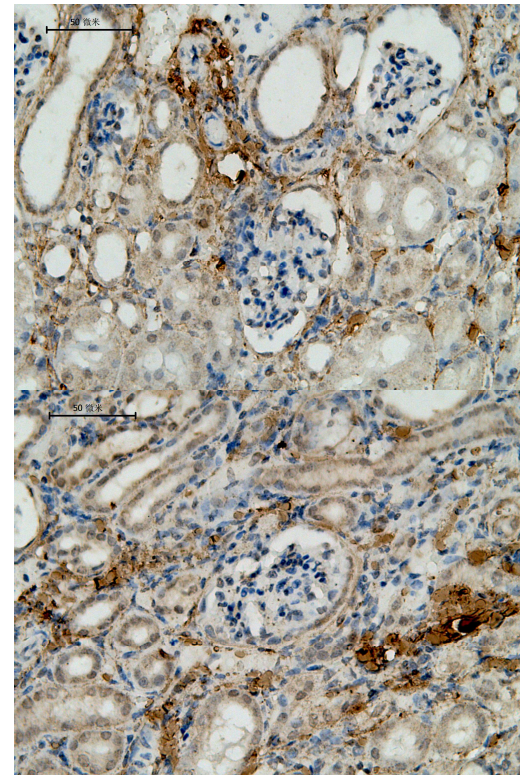

KO

Supplement: S3 File — (PDF) [file pone.0314149.s003.pdf]

## Slide 1
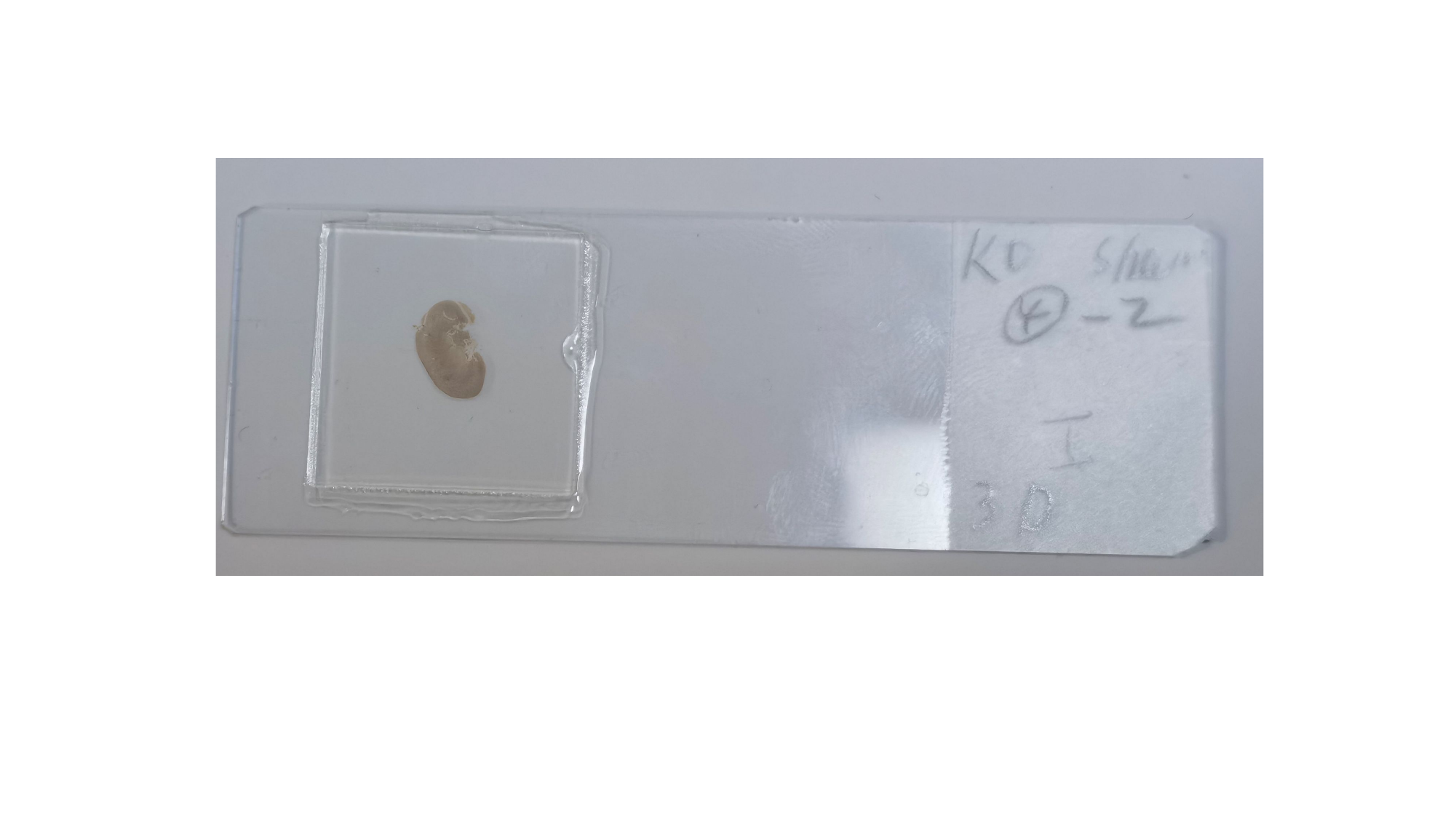

## Slide 2
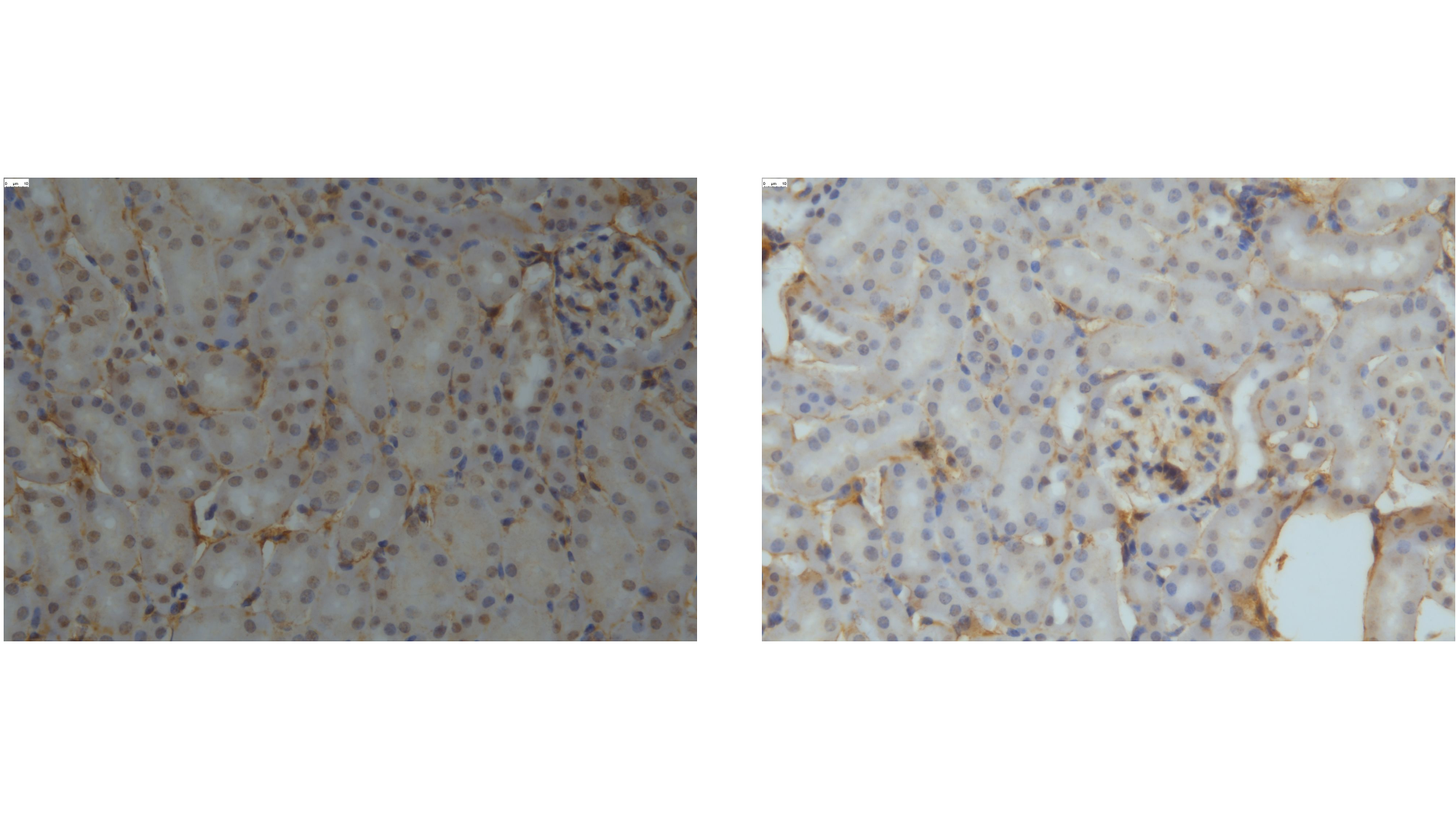

Supplement: S4 File — (PPTX) [file pone.0314149.s004.pptx]

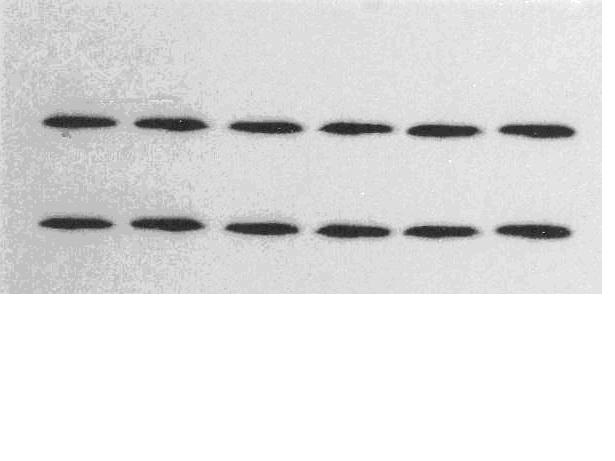

Supplement: S5 File — (JPG) [file pone.0314149.s005.jpg]
